# Non-Invasive Prenatal Testing (NIPT): Reliability, Challenges, and Future Directions

**DOI:** 10.3390/diagnostics13152570

**Published:** 2023-08-02

**Authors:** Siva Shantini Jayashankar, Muhammad Luqman Nasaruddin, Muhammad Faiz Hassan, Rima Anggrena Dasrilsyah, Mohamad Nasir Shafiee, Noor Akmal Shareela Ismail, Ekram Alias

**Affiliations:** 1 Department of Biochemistry, Faculty of Medicine, Universiti Kebangsaan Malaysia, Jalan Yaacob Latif, Bandar Tun Razak, Kuala Lumpur 56000, Malaysia; p113622@siswa.ukm.edu.my (S.S.J.); mlnasaruddin@ukm.edu.my (M.L.N.); nasismail@ukm.edu.my (N.A.S.I.); 2 Department of Surgery, Hospital Batu Pahat, Batu Pahat 83000, Malaysia; fan_pai@yahoo.com; 3 Department of Obstetrics and Gynaecology, Faculty of Medicine and Health Sciences, Universiti Putra Malaysia, Serdang 43400, Malaysia; rimadasril@upm.edu.my; 4 Department of Obstetrics and Gynaecology, Faculty of Medicine, Universiti Kebangsaan Malaysia, Jalan Yaacob Latif, Bandar Tun Razak, Kuala Lumpur 56000, Malaysia; nasirshafiee@ukm.edu.my

**Keywords:** NIPT, prenatal testing, pregnancy, aneuploidy, trisomy, next-generation sequencing, cell-free foetal DNA (cffDNA), obstetrics

## Abstract

Non-invasive prenatal testing was first discovered in 1988; it was primarily thought to be able to detect common aneuploidies, such as Patau syndrome (T13), Edward Syndrome (T18), and Down syndrome (T21). It comprises a simple technique involving the analysis of cell-free foetal DNA (cffDNA) obtained through maternal serum, using advances in next-generation sequencing. NIPT has shown promise as a simple and low-risk screening test, leading various governments and private organizations worldwide to dedicate significant resources towards its integration into national healthcare initiatives as well as the formation of consortia and research studies aimed at standardizing its implementation. This article aims to review the reliability of NIPT while discussing the current challenges prevalent among different communities worldwide.

## 1. Introduction

### 1.1. The Role of Aneuploidy and Trisomy in Congenital Disorders

Classically, humans possess two sets of haploid cells, one from the father and another from the mother, forming euploid cells [1]. Aneuploidy occurs when a person inherits an inaccurate number of haploid cells, either having more or fewer chromosomes than a typical set of 46 chromosomes, and is thus considered a type of chromosomal abnormality [1]. The common cause for aneuploidy is non-disjunction during meiosis I or II, or mitosis, resulting in trisomic or monosomic zygotes, like those associated with Patau syndrome (T13), Edward syndrome (T18), and Down syndrome (T21) [1]. According to the American College of Obstetricians and Gynecologists, chromosomal abnormalities affect 1 in 150 pregnancies [2] and are also a factor in 50% of early pregnancy losses [3]. The risk worsens with increasing maternal age, regardless of singleton or twin pregnancies [2]. Aneuploidies may provoke severe obstetric complications, such as stillbirth, miscarriage, foetal anomalies, and typical facial dysmorphics, along with physical and intellectual disabilities [4]. As foetal aneuploidies are associated with congenital malformation, health organizations and medical committees strongly recommend prenatal screening for foetal anomalies to be performed during the first trimester of pregnancy to mitigate pregnancy complications [4,5].

### 1.2. Cell-Free Foetal DNA: A Discovery with Promising Application

Lo and colleagues proposed the presence of cell-free foetal DNA in maternal blood circulation in 1997 [6]. Cell-free foetal DNA (cffDNA) originates from the placenta and then moves as a result of maternal blood circulation [7]. Initially, placental cytotrophoblasts fuse with syncytiotrophoblasts in order to mature, before being incorporated into maternal circulation through syncytial knots [7]. The breakdown of these knots in maternal circulation sheds foetal DNA, which typically has less than 313 DNA base pairs, as compared to maternal cellular DNA, which displays an average length of 400–500 base pairs [8,9]. This allows easier identification of cffDNA in maternal serum with simple methods like phlebotomy. cffDNA is detected as early as 4 weeks of gestation [10], and the foetal fraction increases to 10–15% of overall maternal plasma from 10 to 20 weeks of gestation [11,12]. However, it is rapidly cleared once the placenta is delivered at the final phase of childbirth. This fact makes cffDNA highly favoured as a biomarker to detect chromosomal aneuploidy, even as early as in the first trimester of pregnancy [13].

### 1.3. Non-Invasive Prenatal Testing (NIPT): A Promising Technique

In addition to its purpose as a biomarker in forecasting obstetrics cases such as pre-eclampsia [14], monogenic disorders [15], and placenta accreta [16,17], cffDNA is invaluable for the analysis and detection of foetal chromosomal abnormalities during the early phase of pregnancy [18,19]. NIPT is a non-invasive prenatal screening technique performed using next-generation sequencing (NGS) to sequence short cffDNA fragments in order to identify the genetic variants that represent chromosomal abnormalities. This screening method was first commercialized in Hong Kong [20], where the research team demonstrated its high sensitivity and specificity when screening for pregnancies at high risk of T21. By using NIPT as the initial screening procedure for high-risk pregnancies with regard to T21, only those who are screened positive in NIPT will proceed with invasive procedures such as chorionic villus sampling or amniocentesis to diagnose T21. This minimizes the unnecessary risk of exposing all pregnant women to invasive diagnostic procedures and the associated complications.

### 1.4. Next-Generation Sequencing (NGS): Surpassing Traditional Molecular Testing

The ability to sequence base pairs of cffDNA would be a laborious task if not for the existence of NGS. Traditional approaches to DNA separation and replication, such as polymerase chain reaction (PCR) and gel electrophoresis, have been part of the Sanger and Maxam–Gilbert methods. Sanger sequencing uses the chain termination method, which produces DNA fragments at different lengths when any dideoxynucleotide (ddNTP) attaches to a DNA sequence to stop the replication [21,22]. The various DNA fragments then undergo gel electrophoresis before sequence analysis.

On the other hand, Maxam–Gilbert sequencing involves the cleavage of different nucleotides in DNA using several different combinations of chemicals [23]. The radioactively labelled fragments are then run on gel electrophoresis, with lanes specified with the cleaved nucleotides before analysis. This technique, also known as the chemical cleavage method, is not favoured over the Sanger method, as the chemicals and radioactive reagents used in the process are hazardous. Moreover, Sanger sequencing can be automated, which makes it suitable for the development of NGS, although larger DNA samples and long hours of work can result in increased costs.

NGS evolves alongside the expansion of advanced sequencing tools with various techniques, such as pyrosequencing and bridge amplification [24]. It reduces the time and costs needed to analyse nucleic acids as compared to conventional methods, hence strengthening research and offering diagnostic potential for NIPT using cffDNA [25].

## 2. Global Introduction of NIPT Via Studies and Consortiums

The global implementation of non-invasive prenatal testing (NIPT) has been facilitated through collaborative research studies and consortiums, with governments and private authorities making significant efforts to integrate such testing into national healthcare programs. Recognizing the immense potential of NIPT in improving prenatal care, these entities have invested considerable resources in promoting its integration into national healthcare programs. By establishing partnerships and fostering research initiatives, they aim to streamline the adoption of NIPT as a routine screening tool, ensuring its accessibility and effectiveness for expectant parents worldwide. This concerted effort underscores the commitment to harness the benefits of advanced genetic testing technologies, ultimately contributing to enhanced prenatal care and improved maternal and foetal outcomes on a global scale. In many countries, NIPT is seen as an promising tool and is in the process of being integrated as a screening technique. In this article, we review some of the prominent NIPT consortiums and programs across continents.

### 2.1. Asia

#### 2.1.1. China

NIPT has existed in China since 2010 [26], but it has recently gained wide attention for its potential in prenatal care. Since the Chinese government’s abolishment of the one-child policy in 2016, older women have expressed concern regarding the risk of carrying a second child in middle age. A study conducted based on a Discrete Choice Experiment in 2020 among Chinese women reported that the participants had a dominant preference for NIPT and suggested it become a part of health insurance coverage in China [27]. The authors also reported participants’ willingness to spend more to ensure the health of the foetus and to take early measures to avoid carrying a child with foetal aneuploidy [27]. Liu and colleagues’ pilot study on implementing NIPT as a first-tier alternative screening test demonstrated its potential use in detecting T13, T18, T21, and sex chromosome aneuploidies, thus suggesting NIPT as a possible replacement for tests used in second-trimester screening, including the traditional serum biochemistry tests used in routine practice [28]. In 2016, the National Health Commission of China published clinical guidelines for the use of NIPT at 12–22 weeks of gestation. Nevertheless, complete government sponsorship of NIPT has not yet been established in China. Some areas, such as Shenzhen, have partial coverage of NIPT with public health insurance. In contrast, in areas such as Zhengzhou, the costs of NIPT are covered by private health insurance or are paid out of pocket [29]. Hong Kong’s public health system has free-of-charge routine prenatal screening for trisomy 21 for women of all ages [30]. As of 2019, NIPT has been incorporated into the publicly funded system as a second-tier screening option for women shown to be at high risk of T13, T18, or T21 through conventional biochemistry analysis [30]. People can opt for NIPT followed by invasive methods like chorionic villus sampling or amniocentesis to have a confirmatory diagnosis or can continue with standard follow-ups without genetic testing. This principle is believed to improve the detection rate for trisomies throughout pregnancy while improving cost-effectiveness.

#### 2.1.2. Japan

NIPT was introduced in Japan in 2013. There was increase in the total prevalence for prenatal testing from 3% in 2008 to 5.3% in 2013 [31,32]. A total of 44,644 pregnant women underwent NIPT by the end of March 2017 [32] There is no comprehensive policy on prenatal testing and NIPT in Japan. In addition, NIPT is primarily paid out of pocket by individuals who need it, despite the cost of NIPT in Japan being among the highest in the world [32]. Thus, NIPT in Japan is only recommended for pregnant women with known high risks of foetal abnormalities [2]. This includes pregnant women with a maternal age of 35 and above, those with detected foetal chromosomal abnormalities via foetal ultrasonography or maternal serum marker tests, or those having a history of a child with a chromosomal aberration. A one-year nationwide clinical study was conducted as a demonstration project to evaluate the screening outcome of NIPT in detecting foetal aneuploidies [33]. The project served to discuss methods to implement NIPT in Japan by testing 7740 women with high-risk pregnancies with regard to aneuploidies, from which 142 positive cases were found. A total of 126 cases went on to confirm the aneuploidies with karyotyping; there were three cases of T21, eight cases of T18, and two cases of T13. The study showed that 98% of cases avoided invasive diagnostic procedures while having a very low false-negative rate of 0.06%.

#### 2.1.3. India

Pregnant women in India prefer NIPT as a prenatal screening option, despite most prenatal care expenditures being paid out of pocket and most procedures being performed at private health institutions [34]. NIPT was first introduced in 2012 in limited regions of India, due to a lack of expertise and the relative unaffordability of the procedure [35]. The Preconception and Prenatal Diagnostic Techniques (PCPNDT) Act of 1994 is responsible for regulating prenatal screening and diagnosis, including NIPT, primarily to prevent parents or physicians misusing prenatal screening techniques for foetal-sex determination and sex-selective abortion [36,37]. NIPT is permitted to be used for the screening of T13, T18, T21, and microdeletions, but foetal sex disclosure to parents is only permitted if genetic abnormalities are detected through prenatal testing [36]. The preference for NIPT to be introduced as second-tier screening is greatly emphasized in some studies [35,38], while others consider it more rational to be used in first-tier screening, as the Medical Termination of Pregnancy Act 1974 allows abortion until 20 weeks of gestation, and NIPT provides opportunities for the early detection of aneuploidies that would assist in decision making regarding termination of pregnancy by the second trimester [37]. Overall, NIPT is slowly being adopted thanks to the PCPNDT Act of 1994.

#### 2.1.4. The Middle East

In Lebanon, there are no official guidelines for the implementation of NIPT; it is recommended as either a first-tier screening or second-tier screening for detecting trisomies or sex chromosome anomalies, depending on the physician and the healthcare centre. In Lebanon, procedures like amniocentesis and maternal serum tests are offered primarily by the private sector, although the public system does cover some of these. A qualitative study indicated that most Lebanese pregnant women or couples having children are more concerned with knowing the genetic condition of their child than with the costs required for NIPT, although, at the same time, they are enthusiastic about NIPT being publicly funded [39]. Similarly, NIPT is available in the private health sector in many other Middle Eastern countries [40,41,42]. The first study on NIPT in Saudi Arabia was published in 2021; NIPT was implemented as a potential choice for first-tier screening to facilitate the detection of high-risk pregnancies with chromosomal aneuploidies in prenatal care [42]. NIPT is also known to be offered in healthcare facilities in Iran [43].

The Israeli National Health Screening framework for prenatal genetic care focuses primarily on combined first-trimester screening (cFTS) and is fully funded by Israel’s Ministry of Health (MOH) or health maintenance organizations (HMOs) [44]. cFTS has an uptake of 60–70% among pregnant women, with appropriate follow-up recommendations given based on the risk assessments performed during the tests [44]. NIPT is currently not a part of the National Health Screening guidelines for detecting chromosomal aneuploidies [44]. In the clinical setting, NIPT is advised as an option for screening for aneuploidies where high risk is determined during the conventional screening of T21 but not as a replacement for invasive diagnostic procedures. Although not funded by the government, some HMOs support the uptake of NIPT by providing up to 75% reimbursement for such services, depending on insurance policies [44].

#### 2.1.5. Southeast Asia

In the Southeast Asia region, governmental bodies have yet to establish guidelines and funding for NIPT, and its application in prenatal screening is predominantly provided by the private sector. In Thailand, Next Generation Genomic Co., Ltd. (Bangkok, Thailand) collaborated with Illumina to launch a new, certified CE-IVD-based NIPT technique to increase the reliability of NIPT for detecting trisomies [45,46]. Another molecular diagnostics and research organization, Sengenics, worked with Lifecodexx AG of Europe to increase the accessibility of their successful PrenaTest^®^ NIPT among pregnant women from Malaysia, Singapore, Brunei, and Vietnam [47]. These efforts served to enlighten women and their families on NIPT as a non-invasive screening method, emphasizing the high level of sensitivity and specificity in detecting pregnancies with chromosomal abnormalities.

### 2.2. Africa

In Africa, NIPT is still considered a novel screening method, as many African countries are in the process of adopting it as part of prenatal screening. According to the South African Society of Obstetrics and Gynecology (SASOG), pregnant women are recommended to undergo NIPT for assessing foetal aneuploidies if they have the financial means, as NIPT is not publicly funded in South Africa [48]. In a similar vein, government initiatives for funding non-invasive prenatal testing (NIPT) are lacking in other African countries. However, private health centres have taken the lead in establishing widespread NIPT services, aiming to educate individuals about this alternative screening option for assessing foetal genetic conditions.

### 2.3. Europe

#### 2.3.1. The United Kingdom

The UK National Screening Committee proposed the use of NIPT for the screening of T13, T18, and T21 in November 2015; the official implementation took place in April 2018 in Wales, September 2020 in Scotland, and May 2021 in England [49,50]. The committee suggested that women with high-risk pregnancies as identified through first-trimester combined screening or second-trimester quadruple screening be eligible for free NIPT [51]. The Nuffield Council of Bioethics concluded that the ethical delivery of NIPT within the Fetal Anomaly Screening Programme is possible provided that accurate information is provided to the public, sufficient education and training is provided for developing medical expertise, and appropriate time is allowed for discussing concerns. In support of this, the Reliable Accurate Prenatal Non-Invasive Diagnosis (RAPID) study suggested the reliability of NIPT as a contingency screening by the National Health Services to minimize the exposure of pregnant women to invasive techniques, while reporting that high levels of informed choice could be achieved if the criteria described by the Nuffield Council were in place [52].

#### 2.3.2. The Netherlands

The Netherlands implemented NIPT as part of a publicly funded foetal aneuploidy screening program in April 2014 via the Trial by Dutch Laboratories for Non-invasive Prenatal Testing-1 (TRIDENT-1) [53], to screen pregnant women at risk of T13, T18, and T21. The government further initiated the TRIDENT-2 study to fund the testing of all pregnant women, regardless of risk exposure, for the screening of foetal aneuploidies [54]. Since the introduction of first-tier NIPT in 2017, the uptake rates were steady at 46% in 2018, while a steep decline for first-trimester combined tests has been observed [55].

#### 2.3.3. Germany

In Germany, the coverage for NIPT for pregnancies with the likelihood of T13, T18, and T21 is suggested to be provided by a publicly funded health insurance system [56]. Since its introduction over a decade ago in Germany, the number of NIPT procedures performed in gynaecological clinics increased from 70 in 2013 to 3000 in 2018 [57]. The current policy on NIPT focuses on individualized decision making with the support of public reimbursement [58].

#### 2.3.4. France

NIPT was initially implemented in France for the screening of T21 only, and occasionally for T13 and T18, with the support of public health insurance. In 2020, the test was further expanded by the private laboratory Cerba to cover the screening of rare aneuploidies like trisomies 2, 8, and 9 as well as large deletions and duplications [59]. At present, the French public system adopts NIPT as an additional screening option that is free of charge for pregnancies involving a high risk of foetal chromosomal abnormalities as determined by the combined first-trimester screening (ultrasound and biochemical markers) [60].

#### 2.3.5. Denmark

In Denmark, NIPT became available as a screening option for chromosomal aneuploidies through the Danish public and tax-financed healthcare system in 2013 [61]. However, in 2017, the Danish Health Authority revised its guidelines on prenatal screening and diagnosis to include NIPT as a standard screening test. The guidelines suggest that all pregnant women should undergo combined first-trimester screening; only high-risk pregnancies as detected by the screening are given the option to proceed with NIPT [61]. Research was conducted using Danish clinical data between 2013 and 2017 to evaluate the use of NIPT before being integrated as a part of the national guidelines [61]. The study reported that in contrast to a high rate of termination observed from a positive invasive test, most pregnant women with a true-positive NIPT result ultimately had live births. Moreover, a minority of women considered NIPT as a risk-free alternative to invasive tests for gaining knowledge regarding genetic conditions to inform their pregnancy decision making.

#### 2.3.6. Belgium

Belgium is the first country to introduce public reimbursement for NIPT as a first-tier screening test and offer it to all pregnant women, while eliminating the necessity for combined first-trimester screening [62]. Currently, serum biochemical analysis is not given priority, but ultrasound tests are still offered as complementary procedures to detect foetal anomalies [62]. Furthermore, the Belgian Advisory Committee on Bioethics specifies that any non-common aneuploidies detected during NIPT must be reported to the patients, accompanied by in-depth genetic counselling [63]. This is to ensure that proper preventive and therapeutic steps are taken to manage these conditions. A two-year consortium involving all Belgian genetic testing centres reported successful implementation of NIPT in first-tier screening; they observed a 52% decrease in the number of invasive procedures conducted and a lower number of T21 live births [64].

#### 2.3.7. Italy

In 2016, the first pilot study validating the use of NIPT in assessing risks for foetal aneuploidies was reported by the Italian Public Health System [65]. The testing accuracies from the study led NIPT to be incorporated into clinical use for detecting T13, T18, and T21. Currently, guidelines for NIPT are specified by the Sistema Sanitario Nazionale (SSN) [66], which is the national public health system, for the screening of foetal aneuploidies among high-risk pregnancies; however, the test is only reimbursed in certain regions, like Toscana and Bolzano [67]. Nevertheless, NIPT is also extensively used in the private sector, leading to an overall uptake of 25% to 50% in the country [68].

#### 2.3.8. Switzerland

Since the formal introduction of NIPT in 2012, there has been a substantial increase in the use of the testing by pregnant women and an overall decrease of 67.4% in invasive prenatal tests, as noted in an early clinical study [69]. According to the Swiss Federal Office of Public Health (FOPH), NIPT has been publicly funded since 2019 by basic health insurance for limited medical reasons, such as to screen for T13, T18, and T21 [70].

#### 2.3.9. Russia

NIPT was initially considered as an additional commercial test to screen for foetal aneuploidies in intermediate- and high-risk pregnancies, as evaluated from conventional screening methods like ultrasonography and serum biochemical markers at weeks 11–14 of gestation. On 13 March 2020, a pilot project consisting of NIPT in the prenatal screening system was conducted by Moscow City Health Department with the collaboration of 23 prenatal care hospitals and one genetic testing laboratory [71]. The project was started in the hope of providing successful adoption of NIPT and establishing official clinical guidelines for NIPT screening at the national level [71]. A preliminary clinical study carried out in the same year to investigate the adoption of NIPT showed it to be effective at screening foetal chromosomal aneuploidies [72]. When the project was completed, the clinical study was repeated to analyse the efficiency of NIPT as a second-line screening test in the first trimester for 12,700 pregnancies [73]. The results showed NIPT as a safe and highly sensitive screening test recommended for all pregnant women in risk groups to detect foetal aneuploidies.

#### 2.3.10. Slovenia

In Slovenia, NIPT is offered only when invasive procedures are contraindicated due to maternal factors such as a pregnancy with a high risk of miscarriage, mothers with transmissible infection to the foetus, and contraction of the uterus [67]. These cases allow NIPT to be offered through public funding despite the test being conducted at full cost to the patient in most private healthcare centres.

#### 2.3.11. Romania

NIPT is offered as a self-financed commercial prenatal screening option in Romania [67]. The first study to report the clinical experience of NIPT among pregnant women in Romania was conducted from the retrospective analysis of 380 NIPT cases from a genetic centre in Western Romania [74]. NIPT was able to demonstrate a high detection rate for autosomal aneuploidies, which led to the suggestion that NIPT be offered as a screening method to all pregnant women [74].

### 2.4. North America and South America

#### 2.4.1. United States

The United States is one of the first countries to routinize NIPT for T21 screening, i.e., since 2011 [75]. While the American College of Obstetrics and Gynaecology recommends NIPT be offered to all pregnant women regardless of their gestational history or risks [67], statistics have shown that an estimated 25% to 50% of pregnant women use NIPT [76], mainly as second-tier screening.

#### 2.4.2. Canada

Canada has adopted NIPT as a publicly funded second-tier prenatal test in three provinces (Quebec, British Columbia, and Ontario) and one territory (Yukon). The Personalized Genomics for Prenatal Abnormalities Screening Using Maternal Blood (PEGASUS) is a national study that was conducted from 2013 to 2017 to provide an evidence-based approach to validating NIPT for its cost-effectiveness in second-tier prenatal screening [77]. The study was a success, as NIPT presented a better decision-making tool for informed choices with regard to prenatal screening, instigated the development of provincial genomic testing technologies, and most importantly, showed that the use of serum screening with conditional NIPT as second-tier screening resulted in the lowest cost for detecting T21, with a rate of $63,139 per case detected [78]. Moreover, this strategy resulted in more than a 90% reduction in invasive procedures such as amniocentesis for the detection of T21. PEGASUS-2 began as a follow-up study in 2018 and ran until 2022, to assess the effectiveness of introducing NIPT as a first-tier prenatal test to screen foetal aneuploidies and other conditions [77].

#### 2.4.3. Mexico

Like most countries, NIPT became widely available in the private sector in Mexico when it was first introduced in 2013 by Natera, a leading US-based organization in prenatal genetic testing, in collaboration with the well-known Mexican fertility institute Médica Fértil, for prenatal genetic screening of chromosomal abnormalities [79]. In 2015, the Genetics Clinic of the Hospital Angeles Lamas employed NIPT for the screening of chromosomal abnormalities and foetal-sex determination among Mexican pregnant women. As predicted, they were able to minimize the number of pregnant women exposed to invasive tests as a result of NIPT. Moreover, the study indicated that NIPT could be a reliable prenatal screening option due to its very high detection rate, specificity, and sensitivity [80]. However, there remain no standard guidelines for NIPT, and most tests are not publicly funded.

#### 2.4.4. Brazil

NIPT received attention among Brazilians when two NIPT test producers from the United States—Ariosa and Natera—partnered with Brazilian biotechnology laboratories to offer the tests to pregnant women in Brazil [81]. This approach was firmly integrated into prenatal genetic screening in the private healthcare sector. Although an expensive procedure, it was welcomed by Brazilian women and achieved significant uptake for numerous reasons, including reducing the expense of frequent ultrasound tests to detect foetal anomalies, ensuring better coverage in detecting all chromosomal abnormalities, not only T13, T18, and T21, and helping the family prepare for the delivery of child with special needs [81].

### 2.5. Oceania

#### 2.5.1. Australia

In Australia, NIPT has been offered to the public since 2012 [82], as a first-tier test for all pregnant women and as a second-tier test for high-risk pregnancies [67]. The prevalence of NIPT has seen a substantial increase, while the frequency of invasive tests has decreased [83]. The test is currently funded out of pocket, with patients seeking reimbursement through a local universal health insurance scheme known as Medicare [84]. Several professional bodies and legal committees have made calls to increase awareness of NIPT among all pregnant women as an available choice for prenatal screening of T21, T13, and T18, though a first-trimester ultrasound test should precede it [85,86]. A total of 25% to 30% of pregnant women are estimated to undergo NIPT in Australia, relating their choice to NIPT’s positive testing experience [84]. Currently, it remains the primary source of diagnosis of T21 during antenatal care [87].

#### 2.5.2. New Zealand

NIPT has been widely accessible in Aotearoa New Zealand since 2013; however, similar to Australia, it is not covered by the public health system [88]. The Royal Australian and New Zealand College of Obstetricians and Gynecologists acknowledge the use of NIPT for screening trisomies and other foetal genetic conditions during pregnancy; however, screening guidelines or specific regulations have yet to be put in place [47]. A report published by Filoche and colleagues outlined the precautions, criteria, and fundamental aspects that should be handled by the National Screening Unit (NSU) and the Ministry of Health when exploring plans to routinize NIPT via the public funding system [88].

## 3. NIPT Is Reliable at Detecting T13, T18, and T21

Numerous studies have evaluated the application of NIPT for detecting T13, T18, and T21 to gain insights into testing accuracy and positive detection. Achieving a better detection rate as compared to the conventional screening methods has been the primary goal of implementing NIPT as part of prenatal screening. A meta-analysis of 37 studies compared cffDNA testing outcomes with foetal karyotype analysis from invasive methods to screen for T13, T18, T21, and other aneuploidies in singleton and twin pregnancies [89]. It was found that the detection rate for T21 was 99.2% for singleton pregnancies and 93.7% for twin pregnancies. Meanwhile, the detection rates for T18 and T13 were 96.3% and 91%, respectively. The study summarized that NIPT provides better detection rates compared to traditional techniques [89]. In a study from Belgium, comparisons among pregnant women who underwent primary NIPT, combined first-trimester screening, or second-trimester triple testing showed that the detection rates for T13, T18, and T21 were the highest in the NIPT screening group. NIPT was also indicated to effectively reduce the invasive tests needed to detect these trisomies by 92.8% [90]. In addition, the study showed a high specificity rate of 99.90% for NIPT in detecting T21 in singleton pregnancies and 99.98% for T18 and T13 [90].

In a retrospective study in South Korea [91], in which all 1055 stored maternal serum samples suspected of foetal aneuploidies underwent NIPT to determine positive testing for trisomies and were further confirmed by karyotype analysis, 108 cases of foetal aneuploidy were identified by NIPT, with a remarkably high sensitivity rate of 100% and specificity of 99.99% for both T21 and T13 [91]. Meanwhile, NIPT had a sensitivity of 92.9% and a specificity of 100% for T18. The overall positive predictive value was 98.1%, showing a range of 90% to 100% in T13, T18, and T21 [91].

In China, a retrospective study conducted in 2020 reported similar results for NIPT outcomes in detecting T13, T18, and T21 among singleton pregnancies [92]. A total of 36,913 pregnancies were involved in NIPT testing, showing 100% sensitivity in determining positive cases for T13, T18, and T21 [92]. The specificity for T13, T18, and T21 was 99.94%, 99.95%, and 99.95%, respectively. Meanwhile, the positive predictive value was the highest for T21, at 84.67%, followed by 58.70% for T18 and 41.94% for T13. Both studies are believed to be significant in establishing NIPT as a highly accurate test for detecting T21, T18, and T13.

Several other studies also reported convincing and high percentages of the sensitivity and specificity of NIPT in detecting T13, T18, and T21 [43,93]. A large international blinded study of 18 955 women detected T21 with 100% sensitivity using NIPT compared to 78.9% using the standard screening method; T18 was detected with 90% sensitivity and T13 with 100% sensitivity by NIPT. Moreover, the specificity for T21, T18, and T13 from NIPT was near perfect, producing rates of 100%, 100%, and 99.9%, respectively. A small study comprising 100 pregnancies found 100% sensitivity of NIPT in detecting T21 [43]. All these studies have shown consistent results, with NIPT having a sensitivity of more than 90% and a specificity of over 99% in identifying T13, T18, and T21; these values are the closest to those produced by invasive testing, suggesting that NIPT could be considered a preferable error-free testing method for the prenatal screening of trisomies.

Another important aspect to be looked at in evaluating NIPT’s ability to detect trisomies is the positive predictive value, which is an indicator of the rate of true positives. A higher positive predictive value represents good reliability for determining positive cases. The positive predictive value from a study in China comprising 17,428 singleton pregnancies using NIPT showed a value of 75% for T13, T18, and T21. An 84.38% positive predictive value was noted for T21, followed by T18 with 61.54% and T13 with 33.33% [94]. A high positive predictive value based on NIPT seems to be most prevalent for T21; for example, a study in Iran found a value of 100% [43].

Another element to note in deducing the effectiveness of NIPT is its ability to reduce the rate of false positives. The findings from Akbari et al.’s study (2018) indicated that NIPT produced a false positive rate of 0.10% for screening T21, which is substantially lower than the 5% rates seen for nuchal translucency ultrasonography and maternal serum marker screening [43]. Meanwhile, in the study by Norton et al. (2015), in which a much larger cohort was involved, the false positive rate was lower, with only 0.05% noted compared to the standard screening method group, which had a rate of 5.4% [93]. An extremely low incidence of false positives for T13, T18, and T21 was also reported in other publications [89,94,95], ranging from 0% to 0.23%.

Twin pregnancies are commonly more complex than singleton pregnancies, particularly due to the presence of the genetic differences in dizygotic twins that can generate only one foetus with a trisomy. However, a multitude of research involving twin pregnancies and NIPT screening has shown perfect or near-perfect results for sensitivity, specificity, and positive predictive value, suggesting that NIPT is also good for the prenatal screening of twins [24,96]. In studies on twin pregnancies, 100% sensitivity of NIPT for detecting T21 was consistently seen [95,97,98,99]. In a study of 25 twin pregnancies that produced seven cases with T21 and one case with T13, no false positives were observed, along with 100% sensitivity and specificity [97]. In addition, 100% specificity for T21 detection with NIPT was reported in a study involving 12 twin pregnancies [98]. Similar findings were seen in the detection of T13, T18, and T21 in 6471 twin pregnancies, i.e., the specificity for T13, T18, and T21 was over 99%; meanwhile, the sensitivity for these aneuploidies was 100% [99]. Similarly, Gill MM’s meta-analysis indicates that NIPT in twin pregnancies can be as reliable and accurate in detecting trisomies as it is for singleton pregnancies [89] (see Table 1).

## 4. Limitations and Challenges

### 4.1. False Positives and False Negatives

Unlike invasive tests such as chorionic villus sampling and amniocentesis, NIPT is still not considered a first-line diagnostic screening method for confirmation of trisomies in pregnancies. The presence of false positives and false negatives reported in studies using NIPT is a stigma for promoting it as a definitive test for diagnosing trisomies. However, the false positives and false negatives generated in studies have been relatively low, including in analyses of large samples [94,101,102,103].

One study detected a false negative rate of 0.09% with NIPT in determining T21 [101]. A prospective study by Xue and colleagues (2020) reported a false negative rate of 0.01% among 81601 pregnancies [102]; nine cases of false negatives were detected, but with size-selection NIPT retesting on these cases, two of the false negatives turned out to be confined placental mosaicism (CPM), and one was a twin pregnancy [102]. The occurrence of false negatives is believed to be due to a low amount of cffDNA in the maternal plasma, influenced by advanced maternal age, high BMI, and early gestation [104,105]. Having shorter cffDNA fragments during DNA extraction and library sequencing would be a good strategy for increasing the cffDNA fraction [106]. Thus, repeating size NIPT (using shorter cffDNA fragments) on false negative cases can identify cases overlooked due to low cffDNA fractions, e.g., CPM or twin pregnancy.

The false positives present in NIPT are always a concern when suggesting NIPT as a first-line choice for prenatal screening. Although the rates are typically less than 1% and are not alarming in most studies, the causes for false positives are not avoidable with technical improvements and can represent future birth complications such as foetal growth retardation, spontaneous foetal reduction, and preterm rupture of membranes [107,108]. CPM is the most common cause [98,109,110]; others, like ‘vanishing twins’ [111,112], maternal copy variants [13,104], and maternal tumours [113,114], are also possible causes. ‘Vanishing twins’ happens when cffDNA floods into the maternal plasma due to necrotic cytotrophoblasts, which in turn causes an influx of foetal DNA in a short time. This phenomenon can last for at least 7-8 weeks but does not last beyond 12-14 weeks of gestation [111]. Tumour-derived cell-free DNA from maternal serum can mask the cffDNA and its chromosomal profile, eventually leading to aberrant NIPT results. Thus, NIPT is a contraindication for pregnant women with malignancies who undergo screening for foetal anomalies [115].

Due to these limitations, NIPT is suggested to be accompanied by other prenatal tests, like ultrasonography, for safer analysis and to avoid mistakes in diagnosis. The capability of early detection and differentiation of false-positive cases from trisomies could also reduce pregnancy complications. Technical and bioinformatic improvements could be made in the future for wider analysis and detection coverage using NIPT [116].

### 4.2. Lack of Expertise

Regarded as patients’ first point of access to information about maternal health and clinical genetics services, obstetricians, gynaecologists, clinical geneticists, and genetic counsellors are essential experts in assisting patients with informed choices and decision making regarding NIPT. However, due to a lack of well-trained clinical experts on prenatal care, patients might not be able to access knowledgeable genetic service providers to provide information on NIPT. In a survey conducted among obstetricians in Texas, it was discovered that all participants were familiar with both NIPT and expanded NIPT [117]. However, 91% of respondents expressed that their understanding was not comprehensive, highlighting the need for ongoing education for healthcare professionals. This emphasis on continuing education aims to enhance the effectiveness of prenatal screening counselling and enable patients to make informed decisions. Furthermore, in-depth interviews conducted with 20 obstetrics experts revealed that inadequate clinical guidance on NIPT contributed to physicians having insufficient skills in introducing the test to their patients [118]. Instances of insufficient NIPT counselling knowledge have been documented, wherein obstetricians and gynaecologists referred pregnant women with abnormal trisomy detected through NIPT for amniocentesis. However, these healthcare providers often struggled to provide adequate interpretations of mosaic trisomy and small supernumerary marker chromosome (sSNMC) as confirmed by amniocentesis [119].

In underprivileged cities or villages where people rely on primary care physicians (PCPs) for medical care, it is a reasonable expectation that PCPs possess appropriate genetics and genomics knowledge and skills to cater to their patients, including providing information on NIPT screening. Unfortunately, there are significant barriers for PCPs, with them citing fewer genetics resources as crucial challenges, including a lack of clinical guidelines, training, and genetics experts [120,121,122]. Such situations must be improved, as patients value opinions and clarifications from clinicians with expertise in NIPT for screening trisomies, considering their inputs as supportive of informed decision making [119,123].

### 4.3. Inadequate Pre-Test and Post-Test NIPT Counselling

During pre-test and post-test counselling on NIPT, pregnant women should also be informed about the test characteristics, screening efficiencies, associated risks, and importance of follow-ups. This allows women to make informed choices on whether they want to opt for the test or choose alternative care. Care providers must show full proficiency in NIPT counselling while assessing for T13, T18, and T21 so that their patients have more assurance and confidence throughout the decision-making process, including before taking the test and after receiving the results [124,125].

Medical or genetics experts that offer pre-test counselling often assume patients have basic knowledge of trisomies like T21 and thus do not thoroughly address the genetic condition. Moreover, some physicians need to inform patients of the other foetal chromosomal abnormalities (including microdeletions) that can also be detected by NIPT [53]. Another misconception provided during pre-test counselling is that NIPT gives high accuracy and thus is proposed as a diagnostic procedure. This mistake was evident from the NIPT screening program in the Netherlands [125]. Furthermore, there is also dissatisfaction with the short counselling time, as many queries regarding the test go unaddressed due to time constraints [123]. On the other hand, too much information provided to patients at one counselling session made patients feel overwhelmed and made it difficult to prioritize information for decision making [126,127].

In a survey among Japanese women regarding NIPT test outcomes, most of the respondents claimed that a lack of information and support after being given the results of the tests promoted negative feelings throughout their pregnancies [128]. They wished for physicians to provide post-test counselling that covered follow-up procedures in the case of receiving a positive result, such as guidance on termination, if needed, or how to prepare for raising a child with anomalies. Thus, professionals should consider these critiques and opinions when establishing a more informative and satisfying counselling session for NIPT.

In the UK, the government assessed health professional counselling skills on NIPT after training sessions; as expected, training helped care providers to be more confident in providing patients with NIPT counselling [129]. Equally, the provider should ensure appropriate counselling techniques. Before introducing NIPT, sufficient data on the patient’s gestation history and reproductive history are essential inputs for the physicians or genetic counsellors [125,130]. It is recommended that practitioners gain consent from patients regarding the intention to be educated and suggesting NIPT as a screening choice for identifying trisomies [128,130]. Overall, a high level of informed choice can avoid biased discussions about NIPT. In addition, post-test follow-ups are mandatory to assist patients with their pregnancy planning, especially in light of positive results [125,131,132].

### 4.4. Culture and Religion

Ethical issues around NIPT have become a concerning topic. Participants in surveys have expressed their concern about the growing reluctance in accepting a child with disabilities and parental rejection of any child that carries genetic abnormalities via termination of pregnancies; they feel that NIPT contributes to this factor, apart from detecting foetal anomalies [128,131]. Cultural differences that exist in societies contribute to a diverse range of views on applying genomic technologies to facilitate decision making. A study in New Zealand described that the Māori believe in the flow of life force (whakapapa) that is interconnected through genealogy and maintained through events like an arranged marriage; the utmost importance of avoiding disruption of this force is emphasized [88]. NIPT is viewed as a contradictory approach towards their belief of whakapapa, especially with regard to abortions following genetic testing [88,133].

Societal acceptance of NIPT could also be greatly influenced by religious views. For example, in a survey conducted among obstetricians in a Muslim country, Pakistan, 94% of respondents felt that NIPT results would significantly affect pregnant women’s decision to continue or terminate pregnancy [134]. The majority of the respondents also agreed that NIPT might increase social pressure on pregnant women to terminate affected pregnancies. This finding suggests that religion could have a great impact on the acceptance of NIPT, because in Muslim countries like Lebanon, for instance, abortion is not allowed unless the mother’s life is at risk [39]. Nonetheless, since NIPT could allow the detection of aneuploidies at a very early stage of pregnancy, NIPT should not be viewed as against Islamic principles, because in Islam, termination of pregnancy is also permissible in early gestational weeks, before the “ensoulment” [135].

It has been reported that some Christian political groups are not in favour of NIPT because of concerns that it would normalize abortions for suspected pregnancies with Down syndrome [136,137]. However, it is interesting to note that findings from a study indicated that health professionals of the Christian faith were more likely to agree that NIPT should be routinely offered to all women, as compared to non-Christians [138]. Nonetheless, it should also be noted that Christian health workers see abortion, in the case of foetal anomalies, as unethical [139].

NIPT appears to be acceptable in the Jewish community; however, it is interesting to note that there is a negative correlation between women who underwent NIPT and their level of religiosity; more religious women are less willing to undergo NIPT [140]. Nevertheless, recent data on the Israeli population, with 74.2% Jewish origin (2013–2019), indicated that a steadily high prevalence of 60–70% of the pregnant population underwent NIPT [44].

Altogether, even though NIPT is still allowed, cultural and religious points of view undeniably have a significant influence on society’s acceptance of NIPT, mainly due to concerns that NIPT would encourage the termination of pregnancies.

### 4.5. Inequality of Accessibility

Healthcare and governmental bodies around the globe have become aware of the significance of NIPT compared to conventional screening methods. Although some countries have approved publicly funded NIPT programs in the primary screening of all pregnant women and introduced NIPT as part of antenatal care [54,56,141], other countries are still hesitant to implement it due to cost restraints. A survey of 28 countries predicted the cost of NIPT to range from USD 350 to 2900, which is considered expensive and limits the widespread use of NIPT [141].

In 2016, the National Health and Family Planning Commission of China published the clinical guideline for NIPT practice, recommending that NIPT be offered throughout the second trimester of gestation [99]. Instead of applying second-trimester screening alone in detecting trisomies, the idea of combining NIPT and the evaluation of maternal age is more productive than China’s current screening strategy in terms of cost-effectiveness and safety [99]. However, the price of NIPT in China could range from USD 202.49 to USD 332.46 in the private sector and is only partially covered by health insurance in most provinces [142]. This situation can become a financial burden for low- to middle-income families when bearing fees under the out-of-pocket scheme. Another study of Chinese women suggested positive support for incorporating NIPT into health insurance coverage in China and emphasized the population’s wish to undergo NIPT [27].

Similarly, restricted insurance coverage is also observed in first-world countries. Many private insurance companies in the US do not cover the initial cost of NIPT for low-risk pregnancies; thus, there is a lack of accessibility with regard to NIPT, which leads some to choose conventional screening methods. Further assessments discovered that women who are covered by public insurance for NIPT to screen for aneuploidies are 3.43 times more likely to opt for the test than those covered by private insurance [143]. The testing is more widely utilized by women from higher-income households, hampering efforts to reduce general exposure to invasive procedures. Interviews with professionals from a combined study in the Netherlands found that pregnant women refrain from NIPT due to financial constraints, especially those from a lower socioeconomic background, who perceive the out-of-pocket contribution to NIPT as a financial burden [125]. This clearly explains why there is a disproportionate relationship between socioeconomic status and access to NIPT.

## 5. Future Directions and Conclusions

NIPT is a rising novel screening technique in medicine, globally acknowledged for its efficiency and embraced by many countries around the world. Despite the aforementioned limitations, NIPT has shown huge potential to be a reliable screening technique, as demonstrated by its high sensitivity and specificity for detecting chromosomal aneuploidies. Although not wholly infallible, NIPT still exceeds conventional screening methods in terms of providing more accurate results in detecting chromosomal aneuploidies.

Governments, including in Southeast Asian countries, should consider routinizing NIPT for detecting T13, T18, and T21 through public funding programs to mitigate disparities among women of diverse socioeconomic backgrounds; meanwhile, health insurance companies can provide testing coverage to assist with reducing the burden of out-of-pocket costs. We can see that the success of public funding for NIPT has benefited women across developed nations like Denmark, the Netherlands, France, and Switzerland in promoting equal access to NIPT for patients [144]. To enhance the informativeness of prenatal screening, it is recommended that physicians or genetic experts who are responsible for NIPT counselling possess sufficient knowledge and skills, enabling them to effectively facilitate the counselling process for pregnant women. Through wider availability of trustworthy information and ease of access to NIPT, future programs and strategies can increase the uptake of NIPT to screen for T13, T18, and T21.

## Figures and Tables

**Table 1:** Summary of clinical studies of NIPT test outcomes.

Authors and Year	Subject	Sensitivity	Specificity	Positive Predictive Value	False Positives
Canick JA et al., 2012 [97]	T21, T18, and T13 screening using maternal plasma of 25 twin pregnancies via massive parallel shotgun sequencing	T13: 100%	T13: 100%	Not reported	0%
		T21: 100%	T21: 100%		
Lau TK et al., 2013 [100]	T21 detection with NIPT among 12 twin pregnancy cases	T21: 100%	T21: 100%	Not reported	Not reported
Norton ME et al., 2015 [93]	15,841 pregnant women that underwent standard screening of nuchal translucency measurement with biochemical tests and NIPT to detect T21, T18, and T13	T13: 100%	T13: 100%	T13: 50%	T13: 2 out of 15,841 pregnancies
		T18: 90%	T18: 100%	T18: 90%	T18: 1 out of 15,841 pregnancies
		T21: 100%	T21: 99.9%	T21: 80.9%	T21: 9 out of 15,841 pregnancies
Gil MM et al., 2015 [89]	Meta-analysis of 37 studies comparing traditional screening methods with NIPT	Singleton pregnancies	Not reported	Not reported	Singleton pregnancies
		T13: 90.3%			T13: 0.23%
		T18: 91.0%			T18: 0.13%
		T21: 96.3%			T21: 0.13%
		Twin pregnancies			Twin pregnancies
		T21: 97.3%			T21: 0.23%
Gerundinho et al., 2016 [65]	195 samples with aneuploid enrichment	T13: 99.9%	T13: 99.4%	Not reported	T13: 2 out of 7 pregnancies
		T18: 99.9%	T18: 99.9%		T18: 0 out of 6 pregnancies
		T21: 99.9%	T21: 98.9%		T21: 2 out of 43
Akbari M et al., 2018 [43]	100 pregnant women underwent NIPT	T21: 100%	T21: 100%	T21:100%	0.10%
Yang et al., 2018 [95]	432 twin pregnancies underwent NIPT to detect chromosomal aneuploidies	T18: 100%	99.53% (combined specificity)	Not reported	1 false positive for T7 and sex chromosome aneuploidy
		T21: 100%			
Kostenko E et al., 2019 [90]	Pregnant women that chose to undergo first-trimester screening or second-trimester screening tests or NIPT	T13: 93.80%	T13: 99.98%	T21: 78.6%	Not reported
		T18: 97.40%	T18: 99.98%		
		T21: 100%	T21: 99.90%		
Kim et al., 2019 [91]	Retrospective analysis of 1055 maternal serum samples	T13: 100%	T13: 99.99%	T13: 90%	2 false positives for T13 and T21 3 false positives for T18
		T18: 92.9%	T18: 100%	T18: 100%	
		T21: 100%	T21: 99.99%	T21: 98.3%	
Lu W et al., 2020 [92]	36,913 women with singleton pregnancies assigned to NIPT	T13: 100%	T13: 99.95%	T13: 41.94%	18 false positives for T13
		T18: 100%	T18: 99.95%	T18: 58.70%	19 false positives for T18
		T21: 100%	T21: 99.94%	T21: 84.67%	21 false positives for T21
Dai R et al., 2021 [94]	17,428 pregnant women that underwent NIPT	Not reported	Not reported	PPV of 75% for T13, T18, and T21 T21: 84.38%; T18: 61.54%; T13: 33.33%	2 false positives for T13
					5 false positives for T18
					5 false positives for T21
Chen Y et al., 2022 [99]	14,574 women with singleton pregnancies and 6471 women with twins that underwent NIPT screening	Sensitivity for T13, T18, and T21 was 100%	Specificity for T13, T18, and T21 was over 99%	T13: 100%	2 false positives for T13
				T18: 75.00%	3 false positives for T18
				T21: 93.75%	2 false positives for T21

## Data Availability

No data were generated from this study; all input was from the literature.
